# Medical Diagnoses Associated with Substance Dependence among Inpatients at a Large Urban Hospital

**DOI:** 10.1371/journal.pone.0131324

**Published:** 2015-06-24

**Authors:** Humberto Choi, Anne Krantz, Jennifer Smith, William Trick

**Affiliations:** 1 Respiratory Institute, Cleveland Clinic, Cleveland, Ohio, United States of America; 2 Division of Pulmonary and Critical Care Medicine, Stroger Hospital of Cook County, Rush University Medical Center, Chicago, Illinois, United States of America; 3 Department of Medicine, Stroger Hospital of Cook County, Rush University Medical Center, Chicago, Illinois, United States of America; 4 Collaborative Research Unit, Department of Medicine, Stroger Hospital of Cook County, Rush University Medical Center, Chicago, Illinois, United States of America; Medical University of Vienna, AUSTRIA

## Abstract

**Background:**

There are limited data on reasons for hospital admission among patients dependent on substances other than alcohol. We compared primary discharge diagnoses for heroin- or cocaine-dependent patients to non-dependent patients.

**Material and Methods:**

We evaluated a cohort of patients admitted to a general medicine service at a public teaching hospital during July 2005-June 2008. Through bedside interviews, we identified patients who had substance-use disorders. We categorized patients by substance used, route of administration, and dependent or non-dependent use. We grouped diagnostic codes (i.e., ICD-9) using Healthcare Utilization Project categories. We excluded HIV-infected patients.

**Results:**

Of 11,397 patients, 341 (3.0%) were dependent on inhalational heroin, 260 (2.3%) on non-injection cocaine, and 106 (0.9%) on injection heroin. Compared to non-dependent patients, inhalational heroin-dependent patients were over three-fold more likely to have been admitted for respiratory diseases (28% vs. 8%, p<0.01); this association was strongest for asthma exacerbation (OR=7.0; 95% CI, 4.7 to 70.4, p<0.01). Of the 225 admissions for an asthma exacerbation, 44 (19.6%) had co-occurrent heroin-dependence. The most frequent diagnostic category among cocaine-dependent patients was circulatory, which was similar to non-dependent patients (22% vs. 21%, p=0.92).

**Discussion:**

There is a strong association between heroin dependence and hospital admission for an asthma exacerbation. Provision of specialized substance-use treatment for inhalational heroin users will be necessary to reduce the frequency of exacerbations and repeat hospital admissions.

## Introduction

In the U.S., substance-use disorders are among the leading preventable causes of mortality and morbidity, and have considerable societal costs.[1,2] The deleterious health consequences attributable to substance-use disorders include the direct toxic effects of substances used, illness related to the method of exposure, and failed adherence to medical therapies. Some of the well-established health consequences include acute intoxication and withdrawal, alcohol-related liver and other gastrointestinal disease, and the myriad infectious complications due to injection drug use, such as endocarditis, skin and soft tissue infections, hepatitis and HIV infections.

In the 1990’s, as inhaled heroin became more prevalent in Chicago, we and others reported the emergence of a new pattern of substance-disease association—that of heroin-related severe asthma requiring ICU admission.[3–4] Another Chicago hospital reported both cocaine and heroin use as a risk for intubation in asthma.[5] There are few if any published data on the linkage between heroin use and asthma exacerbations of lesser severity, not requiring ICU admission.

As part of an effort to evaluate whether hospitalization is an effective opportunity for identifying and intervening on patients who have substance use disorders, our institution implemented routine screening for all inpatients since 2004; initial funding was provided through the Screening, Brief Intervention, and Referral to Treatment (SBIRT) program of the Substance Abuse and Mental Health Services Administration (SAMHSA).[6] All patients are targeted for a bedside interview by trained health counselors, who function independently of the patient’s primary care giving team.

Using substance use data collected for the SBIRT program, we identified a cohort of adult patients admitted to the general medicine service, ascertained their primary discharge diagnosis, and evaluated the association between substance dependence and their primary medical diagnosis. We describe the associations between primary diagnosis and substance dependence and, in particular, the relationship between heroin and cocaine use and asthma.

## Materials and Methods

We evaluated hospital patients ≥ 18 years of age who were admitted to a non-ICU general medicine service at John H. Stroger Jr. Hospital of Cook County, a 464-bed public teaching hospital. We analyzed the initial hospitalization for patients admitted during July 1^st^ 2005 through June 30^th^ 2008; HIV patients were admitted to a separate service and were excluded.

We assessed patients’ substance use history through a structured bedside interview administered by trained health counselors. For all patients, we recorded age, gender, self reported race and Hispanic ethnicity, medical service, and alcohol and drug use. Discharge diagnoses were represented by the International Classification of Diseases 9^th^ Revision (ICD-9) coding schema, which included a designation for the primary discharge diagnosis. Since there are thousands of diagnostic codes, we aggregated individual ICD9 codes into categories using the Healthcare Cost and Utilization Project’s (HCUP) clinical classification software.[7] We used the multi-level coding schema that places ICD9 codes into 18 categories; we report 16 categories as no patients were in the categories for “Conditions related to the perinatal period” or “Residual codes”.[8] In the HCUP classification, respiratory infections, such as pneumonia or influenza, are categorized under diseases of the respiratory system; codes related to substance- and alcohol-related disorders are categorized under the broad category Mental disorders; and the category “symptoms, signs and ill-defined conditions” includes ill-defined conditions such as syncope, abdominal pain, social admission, and malaise and fatigue.

From our bedside interviews, we categorized patients’ substance use into the following three categories: “low risk”, i.e. no illicit drug use in the past 3 months and alcohol use below the threshold for hazardous use as defined by the National Institute on Alcohol Abuse and Alcoholism (NIAAA) guidelines; “at risk”, i.e. alcohol or recent illicit drug use without criteria for dependence; or, “dependent”, i.e., alcohol or illicit drug use with criteria for dependence. We considered patients as substance dependent if they affirmed three or more consequences of use when queried using a modified version of the Texas Christian University (TCU) drug screen, which examines nine potential deleterious social and health characteristics of use.[9,10] Since we determined the presence or absence of substance dependence for each patient, rather than for each substance, we considered a patient dependent for the substance they reported as their worst problem substance. For this analysis, we focused on the comparison between patients who had low risk use and those who had dependent use. Since we believed the distribution of diagnoses would differ for patients who reported injection drug use (either by intravenous or subcutaneous administration) rather than only inhalational use, we created a separate category for injection drug users. As patients could be admitted multiple times and because substance dependence may be associated with re-admission, we performed a patient-level analysis, including only a patient’s initial hospitalization during the study period. The number of patients reporting substance dependence to marijuana, amphetamines, benzodiazepines or hallucinogens was too infrequent to permit precise estimates and these were omitted from analysis.

Counselors entered the data collected from their bedside interviews onto a standardized paper-based data collection form, which was entered into an Access (Microsoft Inc., Redmond, WA) database and then transferred to a Microsoft SQL Server (Microsoft Inc., Redmond, WA) database. Bedside interview data were joined to an institution-wide administrative database that contains discharge ICD-9 diagnoses for each admission. [11] For each comparison of substance-dependent patients, non-dependent patients were considered as the referent group. To adjust for potential confounders for the association between substance dependence and specific diagnoses (e.g., asthma), we constructed logistic regression models that included additional covariates, such as age, gender, race, smoking status and cocaine use. We performed statistical significance testing using the t-test for continuous variables and using the chi-square test for categorical variables, all p-values were two tailed. All data manipulation and analyses were performed using Stata version 10 (Stata Inc., College Station, TX) or SPSS 17 (SPSS Inc., Chicago, IL) software. The study presented minimal risk and qualified for a waiver of consent. The study presented minimal risk and involved collecting and analyzing only existing data by review of medical records, and it was not practical to obtain written consent due to the large number of subjects. The Institutional Review Board at Cook County Health and Hospitals System approved this study and waived the need for written informed consent from the participants (IRB #08–155).

## Results

Of 12,947 patient interviews, we successfully matched admission records for 11,397 (88%) of patients. The mean age was 51 years, over half were male, and a majority were African-American (Table 1). Alcohol was the most common substance used, followed by cocaine, marijuana, and heroin (Table 1). Over half of patients who reported heroin use (n = 590) were categorized as dependent (60%), whereas just one third of the patients who reported cocaine (n = 775) use and 40% of patients who reported at-risk alcohol use (n = 1516) were categorized as dependent. Marijuana use was common, but rarely identified as the substance of greatest concern to the patient. Among dependent patients, alcohol dependence was most common (n = 720; 6.3%), followed by inhalational heroin (n = 341; 3.0%), non-injection cocaine (n = 260; 2.3%), and injection heroin use (n = 106; 0.9%). Among heroin-dependent patients, nasal inhalation was most common (n = 329, 73.6%), followed by injection (n = 106; 23.7%) and smoking (n = 12; 2.7%).

**Table 1:** Characteristics of general medicine inpatients included in the study, July 1, 2005 through June 30, 2008.

Characteristics	Total participantsn = 11397	
Age(years), mean (SD)	51	14.9
	n	%
Gender, female	4695	41.2
***Race***		
African-American	6531	57.3
Hispanic	2545	22.3
White	1703	14.9
Asian	495	4.3
Other	123	2.3
***Substance use assessment***		
Low health risk	8427	73.9
Current tobacco use	3854	33.8
***At risk use last 30 days***		
Alcohol	1516	13.3
Cocaine^a^	775	6.8
Marijuana	620	5.4
Heroin^a^	590	5.2
***Dependent***		
Alcohol	720	6.3
Inhalational heroin	341	3.0
Non-injection cocaine	260	2.3
Injection heroin	106	0.9
Marijuana	20	0.2

^a^ Injection and/or non-injection drug use

The demographic characteristics and pattern of substance use associated with each category is shown in Table 2. Patients in the dependent categories, regardless of substance, had a mean age of 10 or more years younger than low-risk patients (P<0.001). Most patients with heroin- or cocaine-dependency were African-American (94.1% and 86.9%, respectively), where as those with injection drug use were disproportionately white. The intensity of substance use was highest among dependent users who reported injection or inhalational heroin use (mean of 22 days over the last 30 days). As we previously reported, the prevalence of current smokers and heavy smoking (> 14 cigarettes per day) was higher among the dependent groups compared to the low-risk group. [12]

**Table 2:** Characteristics of patients, stratified by substance use and type of substance used.

	Low risk use		Dependent use					
	n = 8427		*Inhalational heroin* n = 341		*Cocaine* n = 260		*Injection heroin* n = 106	
	n	%	n	%	n	%	n	%
Age (mean, SD)	53 ± 15		46 ± 8		46 ± 9		43 ± 12	
Gender, female	3958	47	119	34.9	92	35.4	40	37.7
***Race***								
African-American	4627	54.9	321	94.1	226	86.9	52	49.1
Hispanic	2073	24.6	10	2.9	16	6.2	20	18.9
White	1182	14	9	2.6	17	6.5	34	32.1
Asian	466	5.5	0	0	0	0	0	0
Other	10	0.1	1	0.3	0	0	0	0
***Frequency of drug use in 30 days (mean*, *SD)***								
Alcohol	12.5 ± 12		5.3 ± 9		7.2 ± 9.5		3.5 ± 8.3	
Cocaine	--		5.6 ± 9.7		11 ± 9.6		6.9 ± 10.6	
Heroin	--		22 ± 9		0.9 ± 4		22 ± 10	
Marijuana	--		0.5 ± 2.9		1.1 ± 4.1		0.6 ± 3.8	
***Tobacco use***								
Current use	1918	22.7	287	84.2	201	77.4	93	87.7
> 14 cigarettes per day	400	4.7	89	26.1	50	19.2	48	45.3

**Fig 1:**
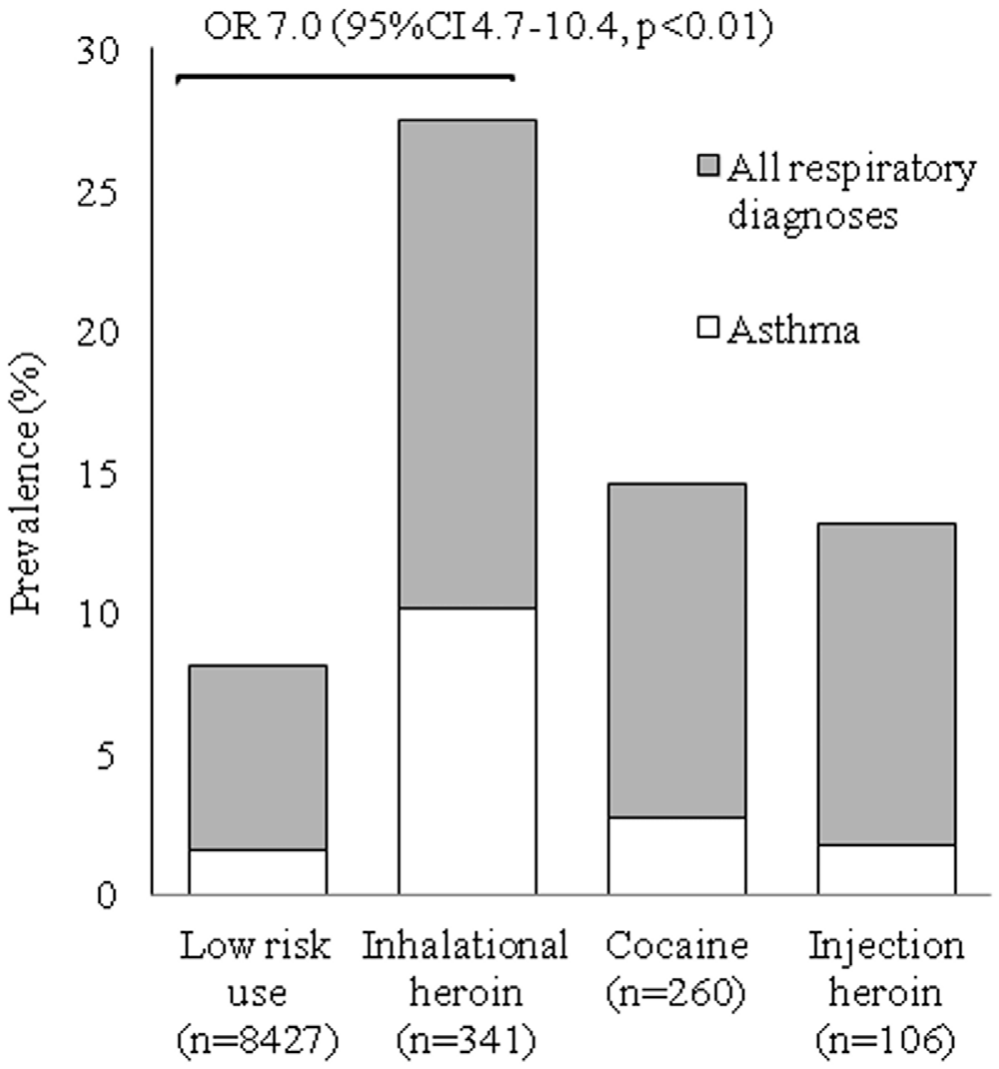
Prevalence of ICD-9 discharge diagnoses that represent all respiratory diseases (gray) and asthma (white). Odds ratios of asthma in drug dependent patients compared to low risk drug use: inhalational heroin (OR 7, 95% CI 4.7–10.4, p<0.001), cocaine (OR 1.7, 95% CI 0.8–3.7, p = 0.20) and injection heroin (OR 1.2, 95% CI 0.3–4.9, p = 0.68).

**Fig 2:**
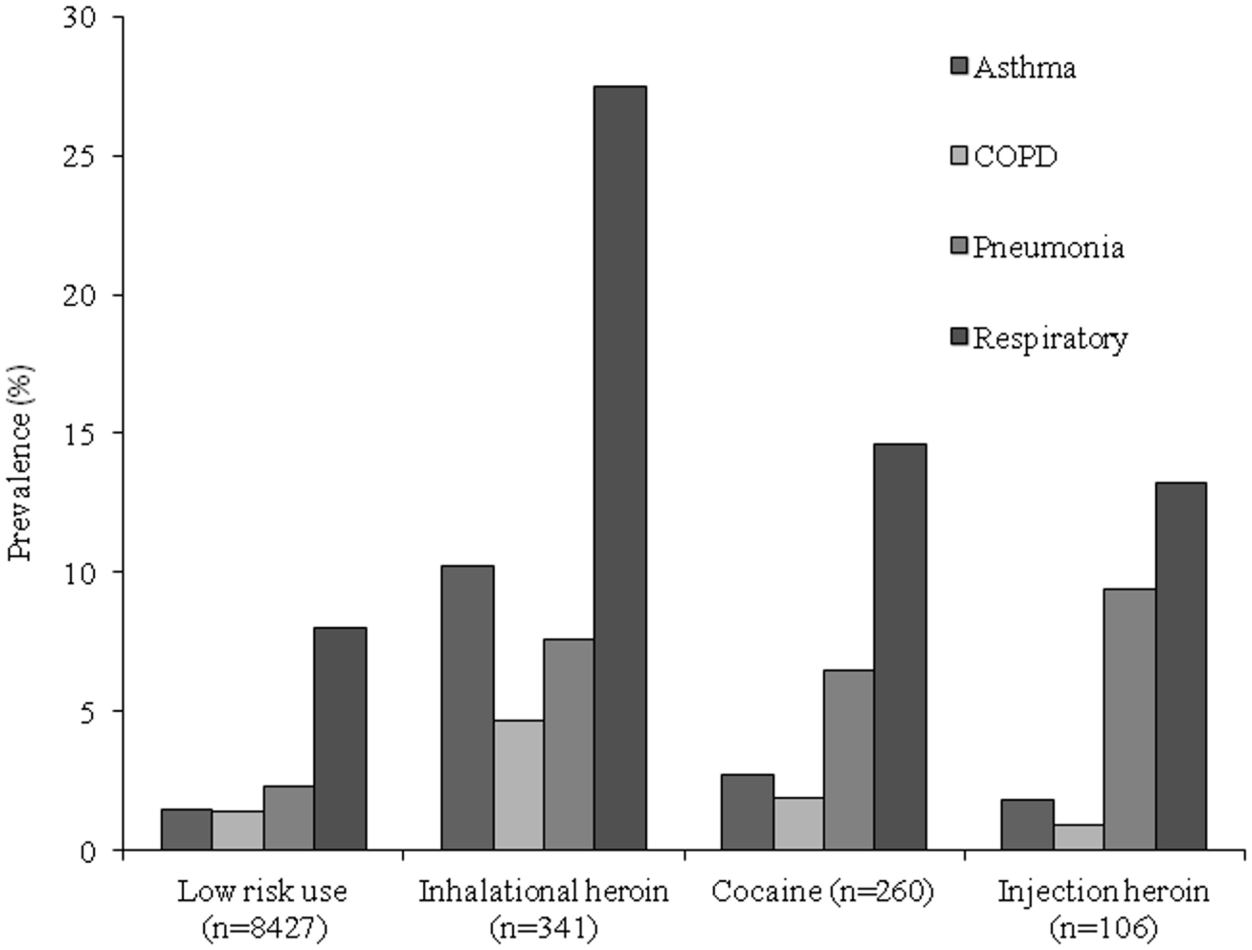
Prevalence of asthma, COPD, pneumonia and respiratory diagnoses among low risk and dependent drug use patients.

**Fig 3:**
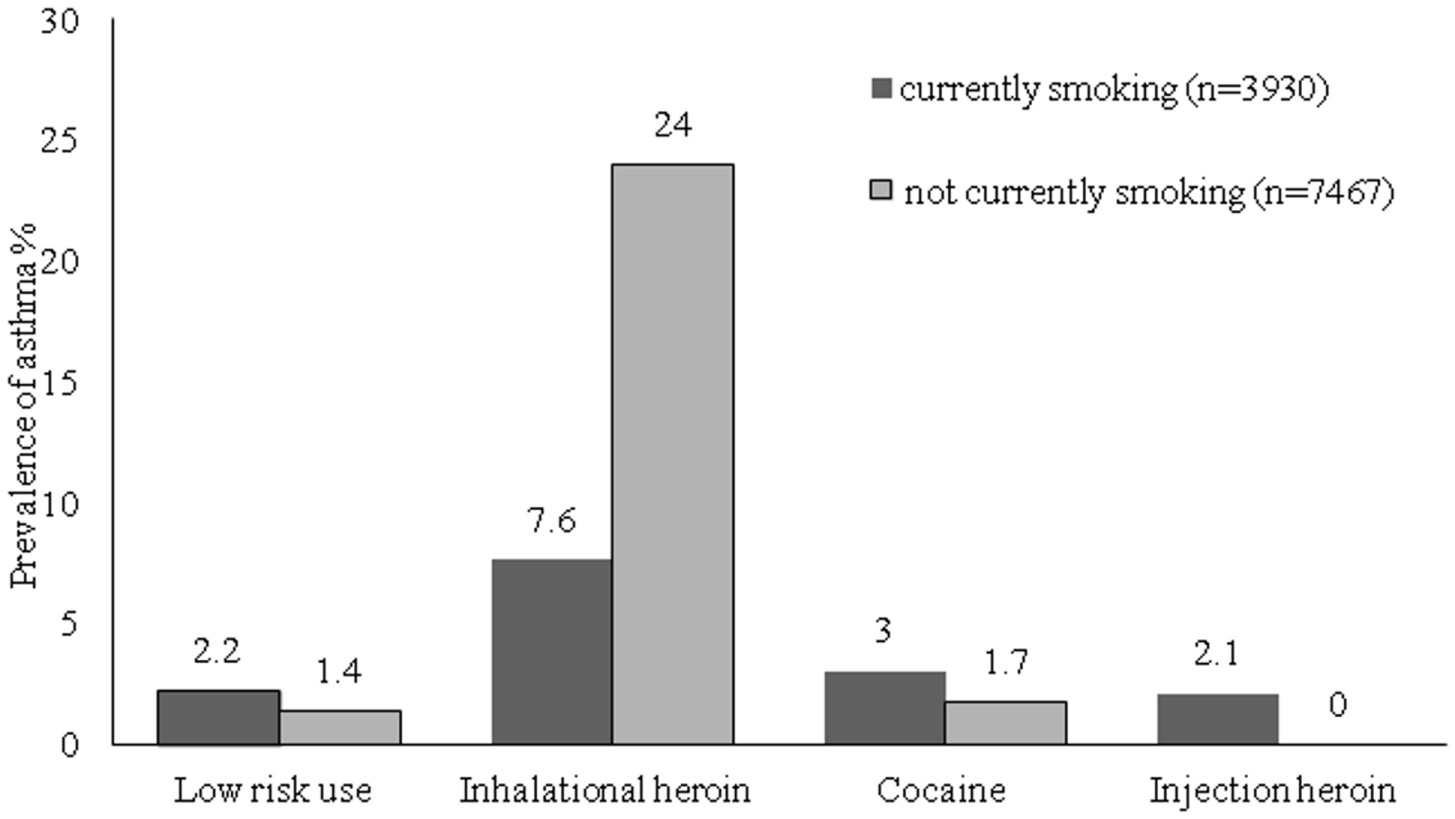
Prevalence of asthma exacerbation among patients with low risk and dependent drug use stratified by tobacco smoking status.

The distribution of primary discharge diagnoses varied by category of substance use (Table 3). Among low-risk patients, the most common discharge diagnoses were diseases of the circulatory system (21%) and neoplasms (14%). For those with alcohol dependency, mental diagnoses were most common, followed by digestive diagnoses, as would be expected, both of which were elevated compared to low-risk patients. Compared to low-risk patients, inhalational heroin-dependent patients were significantly more likely to be admitted for respiratory diseases (28% vs. 8.1%, p<0.01); injuries and poisoning (9.7% vs. 5.9%, p<0.01); mental disorders (6.5% vs. 1.1%, p<0.01); and for “symptoms, signs, and ill-defined conditions” (10.6% vs. 7.4%, p<0.05). The most frequent diagnostic category among cocaine dependent patients was circulatory-similar to non-dependent patients (22% vs. 21%, p = 0.92). However the cocaine-dependent group was 8 years younger than low-risk patients (p<0.01). Compared to low-risk patients, cocaine-dependent patients were more likely to be admitted for respiratory diseases (14.6% vs. 8.1%, p<0.01); “symptoms, signs, and ill-defined conditions” (15% vs. 7.4%, p<0.01); injuries and poisoning (10.1% vs. 5.9%, p<0.01), infectious diseases (5.1% vs. 2.1%, p<0.01); and mental disorders (3.8% vs. 1.1%, p<0.01). Injection heroin-dependent patients were more likely to be admitted due to diseases of the skin and subcutaneous tissue (e.g., cellulitis) (21.7% vs. 4.9%, p<0.01), or mental disorders (17.4% vs. 1.1%, p<0.01) compared to the low-risk group. When we explored the association between inhalational drug use and respiratory diseases, we found that the relationship was particularly strong for inhalational heroin use and admission for an asthma exacerbation (Fig 1). We also found a dose-response association: patients who reported daily inhalational heroin use over the prior 30 days were significantly more likely to be hospitalized for an asthma exacerbation compared to heroin-dependent patients who used heroin less frequently (OR 2.3; 95% CI, 1.3 to 4.1, p<0.01). The prevalence for all three of the most common respiratory diagnoses (asthma, COPD, and pneumonia) was higher for heroin users than low-risk users (Fig 2). Since smoking was a possible strong confounder for the association between asthma and heroin dependence, we evaluated the prevalence of asthma after stratifying by smoking status; we found that the prevalence of asthma was highest for non-smoking inhalational heroin dependent patients (Fig 3).

**Table 3:** Prevalence of ICD-9 groups among patients with low risk and dependent drug use.

ICD-9 groups	Low risk use n = 8427		Inhalational heroin n = 341		Cocaine n = 260		Injection heroin n = 106	
	n	%	n	%	n	%	n	%
Infectious and parasitic diseases	181	2.1	13	3.8	13	5.0^b^	2	1.9
Neoplasms	1147	14.0	17	5.0^a^	5	1.9	1	0.9^a^
Endocrine, nutritional and metabolic diseases, and immunity disorders	481	5.7	8	2.3^a^	3	1.2^a^	0	0^a^
Diseases of the blood and blood-forming organs	247	2.9	8	2.3	5	1.9	1	0.9
Mental disorders	95	1.1	22	6.5^b^	10	3.8^b^	17	16.0^b^
Diseases of the nervous system	175	2.1	3	0.9	1	0.4	2	1.9
Diseases of the sense organs	66	0.8	1	0.3	2	0.8	1	0.9
Diseases of the circulatory system	1806	21.4	43	12.6^a^	58	22.0	9	8.5^a^
Diseases of the respiratory system	679	8.1	94	28.0^b^	38	14.6^b^	14	13.2
Diseases of the digestive system	1110	13.2	26	7.6^a^	21	8.1^c^	6	5.7^a^
Diseases of the genitourinary system	444	5.3	7	2.1	12	4.6	3	2.8
Diseases of the skin and subcutaneous tissue	410	4.9	20	5.9	14	5.4	24	22.6^b^
Diseases of the musculoskeletal system and connective tissue	378	4.5	6	1.8^c^	10	3.8	5	4.7
Symptoms, signs and ill-defined conditions	621	7.4	36	10.6^c^	39	15^b^	11	10.4
Injury and poisoning	494	5.9	33	9.7^b^	26	10^c^	8	7.5
External causes of injury	29	0.3	2	0.6	2	0.8	1	0.9

Statistical testing performed using the low risk category as the comparator group.

^a^ p<0.05 higher prevalence among low risk patients.

^b^ p< 0.01 higher prevalence among dependent patients.

^c^ p<0.05 higher prevalence among dependent patients

By multivariable analysis, after adjusting for age, gender, smoking status, African-American race and cocaine use, inhalational heroin use remained significantly associated with a diagnosis of asthma (adjusted OR = 2.1, 95% CI 1.5 to 2.8). To minimize the likelihood that COPD misdiagnosed as asthma contributed to our findings, we performed the same analysis on patients < 50 years of age; the association between inhalational heroin dependence and asthma remained strong (adjusted OR = 2.0, 95% CI 1.4 to 3.0). In contrast, after adjusting for the factors above and heroin use, cocaine was not associated with asthma.

## Discussion

We found that the reasons for hospitalization on a general medicine service varied based on presence or absence of a substance use disorder, the substance used, and the route of use. Most evident was the strong association between respiratory diagnoses, especially asthma, and dependence on inhalational heroin. Other findings have been previously well described and were expected, such as the most common reasons for admission among alcohol-dependent patients (digestive or mental [includes withdrawal]) or heroin-dependent patients who were injection users (skin infections). [13,14] Among cocaine-dependent patients, the most frequent cause for hospitalization was circulatory, for which the prevalence was similar to the low-risk group; however, cocaine-dependent patients were a mean of eight years younger than the low-risk group. In fact, patients with substance use disorders were a mean of 10 years younger than those with no use or low-risk use.

Our findings extend the previously observed association between inhaled heroin and ICU asthma to patients with a less severe exacerbation. [3–4] Several features of data now strengthen our understanding that the association between heroin inhalation and asthma is a causal one, and not simply the co-occurrence of two epidemics in the same population. We observed a dose response relationship, i.e., daily use of heroin was more likely to have been associated with admission for asthma compared to less frequent use. While persons with asthma and coincident substance dependence may have a higher likelihood of hospital admission because of poor adherence to disease self-management, this association did not hold up in patients with other substance use disorders.[15] Finally, the finding persisted even after adjusting for race. Thus, the evidence suggests a causal relationship between inhalational heroin use and asthma exacerbation, which includes the strength of association, a dose-response relationship, consistency with prior reports, and, as follows, potential pathophysiologic mechanisms.[3]

Previous reports of bronchospasm due to heroin or morphine inhalation have suggested mechanisms by which this may occur: the bronchoconstrictive effect of morphine (a heroin metabolite); mast cell degranulation, and allergic.[16–19] Others have proposed direct thermal injury from drug use by smoking but in our study very few smoked heroin. In addition, there may be drug additives and contaminants that may be contributory, and more than one mechanism may be relevant for different patients.

To what extent can substance use be considered contributory to the well known inner-city asthma epidemics? In a study of asthma deaths in Cook County, where our hospital is located, for 32% of asthma-related deaths there was evidence for recent substance use, and in a separate study, 18% of decedents had a history of drug abuse and 13% had toxicology screens positive for illicit drugs. [20,21] Despite these and other findings, we caution against overattribution of substance use as a significant driver of overall severity in the well-known asthma epidemics of Chicago and other cities. Asthma in vulnerable communities is first a pediatric epidemic, for which multiple household and environmental risks have been identified; heroin use, even among adults in vulnerable communities, is very uncommon; and, finally, the rate of asthma-related hospitalization and death has declined steadily in Chicago since the publication of the first asthma guidelines and concomitant rise in the use of inhaled steroids. [22–24] Thus it is likely that heroin users represent only a small subset of patients at risk for severe asthma.

Cocaine—dependent patients were most likely to be admitted for disorders of the circulatory system. In comparison to low-risk patients, the prevalence of circulatory disorders was not different in the cocaine-dependent group; however, cocaine-dependent patients were on average 8 years younger than low-risk patients. The younger age of admission for patients who were cocaine-dependent may reflect the occurrence of vascular disease at a younger age or perhaps non-specific chest pain syndromes, which are categorized as circulatory disorders. Cocaine-dependent patients also were more likely to be admitted for respiratory diseases; however, these admissions were much less common then for heroin-dependent patients. Our findings that skin and soft-tissue infections were more common among injection heroin users was expected and has previously been reported.[14]

To our knowledge, these data represent the largest published analysis of the reason for medical hospitalization according to the presence of drug use disorders, as established by systematic assessment. However, our study has several limitations. We determined whether a patient met criteria for substance dependence and attributed the dependence to the substance they identified as their biggest problem substance; our method did not allow for patients to be in several categories. Because substance dependency was assessed by patient responses to face-to-face interviews, there may have been underreporting of substance use or minimization of the impact of substance use. However, we would not expect that vary by medical diagnosis, and misclassification of patients’ substance use history likely would have resulted in reduced estimates of the differences in the prevalence of diagnoses. Finally, our findings are representative of patients admitted to a public hospital in an urban setting where inhalational heroin use is common, and may not reflect other settings.

A better understanding of substance use and reason for hospital admission has significant implications for the care of persons with substance use disorders and may lead to increased knowledge of treatment needs. The recognition of an illness that is substance related may motivate both patient and physician to address the underlying substance dependency. Clinicians should include an evaluation for substance use for all hospitalized patients; however, they should be particularly aware of the association between inhalational heroin use and asthma. Identifying heroin dependence and referring these patients to specialized treatment centers should be one component of strategies designed to reduce asthma morbidity and mortality.

## Supporting Information

S1 File Dataset. (ZIP)
